# Recruiting a new strategy to improve levan production in *Bacillus amyloliquefaciens*

**DOI:** 10.1038/srep13814

**Published:** 2015-09-08

**Authors:** Jun Feng, Yanyan Gu, Yufen Quan, Wei Zhang, Mingfeng Cao, Weixia Gao, Cunjiang Song, Chao Yang, Shufang Wang

**Affiliations:** 1 Key Laboratory of Molecular Microbiology and Technology of the Ministry of Education, Nankai University, Tianjin 300071, China; 2 Department of Microbiology, College of Life Science, Nankai University, Tianjin 300071 China; 3 State Key Laboratory of Medicinal Chemical Biology, Nankai University, 94 Weijin Road, Tianjin 300071, China; 4 Department of Chemical and Biological Engineering, Iowa State University, Ames, Iowa 50011, United States

## Abstract

Microbial levan is an important biopolymer with considerable potential in food and medical applications. *Bacillus amyloliquefaciens* NK-ΔLP strain can produce high-purity, low-molecular-weight levan, but production is relatively low. To enhance the production of levan, six extracellular protease genes (*bpr*, *epr*, *mpr*, *vpr*, *nprE* and *aprE*), together with the *tasA* gene (encoding the major biofilm matrix protein TasA) and the *pgsBCA* cluster (responsible for poly-γ-glutamic acid (γ-PGA) synthesis), were intentionally knocked out in the *Bacillus amyloliquefaciens* NK-1 strain. The highest levan production (31.1 g/L) was obtained from the NK-Q-7 strain (Δ*tasA*, Δ*bpr*, Δ*epr*, Δ*mpr*, Δ*vpr*, Δ*nprE,* Δ*aprE* and Δ*pgsBCA*), which was 103% higher than that of the NK-ΔLP strain (Δ*pgsBCA*) (15.3 g/L). Furthermore, the NK-Q-7 strain also showed a 94.1% increase in α-amylase production compared with NK-ΔLP strain, suggesting a positive effect of extracellular protease genes deficient on the production of endogenously secreted proteins. This is the first report of the improvement of levan production in microbes deficient in extracellular proteases and TasA, and the NK-Q-7 strain exhibits outstanding characteristics for extracellular protein production or extracellular protein related product synthesis.

Microbial levan, one of the two main types of fructan biopolymers, is mainly polymerized via β-(2 → 6) bonds^1^ and has been isolated from Gram-negative bacteria, Gram-positive bacteria and some fungi^2^. Levan has many favourable properties, and is used in variety of industrial applications including in foods, cosmetics and pharmaceuticals^3^,^4^,^5^.

Previous work has revealed that microbial levan is synthesized in the medium by the secreted levansucrase (EC: 2.4.1.10) from the sucrose substrate^6^. Microorganisms synthesize higher-molecular-weight levan at the beginning of fermentation, after which the molecule is hydrolyzed to lower-molecular-weight levan products in the presence of the β-2,6-fructofuranoside linkage-hydrolyzing enzyme, levanase^5^.

*Bacillus amyloliquefaciens* NK-1 has the ability to co-produce γ-PGA and levan during fermentation. The *pgsBCA* genes (responsible for γ-PGA synthesis) deletion strain *B. amyloliquefaciens* NK-ΔLP can produce high-purity levan, with the highest titer observed being 14 g/L^7^. The molecular weight of levan obtained from the NK-ΔLP strain is around 5 kDa, which is much lower than other reported levan products^8^. The *Bacillus amyloliquefaciens* strain was isolated from fermented food^9^, thus its levan product was supposed to have the potential to be the dietary supplements^10^. However, the low levan production by this strain is unable to meet industrial demands. Therefore, further strain improvement through metabolic engineering is required.

Most of the strategies applied thus far to enhance levan production have been limited to medium optimization or fermentation process improvement^11^,^12^, and only a few metabolic engineering strategies have been utilized to improve levan production. Senthilkumar *et al.*^13^ studied the effect of the disruption of the *Zymomonas mobilis* extracellular sucrase gene (*sacC*) on levan production. The *sacC* gene mutant strain showed three-fold higher levansucrase (SacB) activity than the wild-type strain and the levan titer increased from 15.5 g/L to 21.2 g/L. Similar genetic modification was a performed in *Lactobacillus reuteri*, demonstrating that disruption of the sucrose phosphorylase gene *scrP*, (encoding a sucrose hydrolysis enzyme), also increased levan production^14^. Shida *et al.*^15^ studied the effects of disrupting the levanase gene *sacC* on levan production in *Bacillus subtilis* 327UH strain. The results showed that there was no difference in the obtained levan yield between the *sacC*-deficient strain and the wild-type strain; however, the polymerization degree of levan obtained from the *sacC*-deficient strain was approximately three times higher compared with that from the wild-type strain. Over-expression of the *sacB* gene is another method for improving levan production. Ananthalakshmy *et al.*^16^ expressed the *sacB* gene by using the pLSD19 plasmid in the sucrase mutant *Zymomonas mobilis* Zsuc1 strain and found that levan production increased from 5 g/L to 10.7 g/L compared with the wild-type strain.

In the current study, we proposed a new strategy for metabolic engineering of the *B. amyloliquefaciens* NK-1 strain to improve levan production. TasA is the main protein in biofilm matrix^17^,^18^. The extracellular matrix surrounds the cells and might block the secretion of levansucrase. Previous works demonstrated that the *tasA* gene deletion strain could only form deficient biofilm^17^. The *tasA* gene was deleted in this work to determine its effects on levan production. *Bacillus* can produce high level of extracellular proteases to degrade the extracellular protein for cell use^19^. We speculated that deleting protease genes can expect to find other extracellular proteins more stable. Thus, we decided to delete the extracellular protease genes to improve levan production. In our previous work, we found that NK-1 strain could co-produce γ-PGA and levan simultaneously; moreover, their purification procedures are similar. Thus, *pgsBCA* genes deletion may increase levan purity as well as its production. A schematic of this proposed genetically engineered metabolic pathway of *B. amyloliquefaciens* NK-1 is shown in Fig. 1. We aimed to improve levan production by carrying out the above-mentioned three tasks: (1) delete the *tasA* gene to make the biofilm formation deficient; (2) delete six extracellular proteases genes *bpr*^20^,^21^, *epr*^22^, *mpr*^23^,^24^, *vpr*^25^, *nprE*^26^ and *aprE*^27^,^28^, to decrease the degradation of the levansucrase; and (3) detete the *pgsBCA* cluster to block the γ-PGA synthesis pathway and obtain a higher purity and yield of the levan product. The final engineered NK-Q-7 strain could produce 31.1 g/L levan in flask, which was 103% higher compared with the production in the NK-ΔLP strain.

## Results

### Construction of marker-less gene deletion mutants

In this work, we sought to improve levan production in a *B. amyloliquefaciens* strain by deleting the *tasA* gene, extracellular protease genes and the *pgsBCA* cluster. For the preferential effect behavioral test on γ-PGA production, we first deleted the *tasA* gene and then sequentially deleted the six extracellular protease genes *bpr*, *epr*, *mpr*, *vpr*, *nprE* and *aprE* in the NK-1 strain. The target strains were designated NK-P-X (X = 1–7). Next, the γ-PGA synthetase cluster *pgsBCA* was deleted from the NK-P-X strains, and the resultant γ-PGA-deficient strains were designated NK-Q-X.

**Figure 2:**
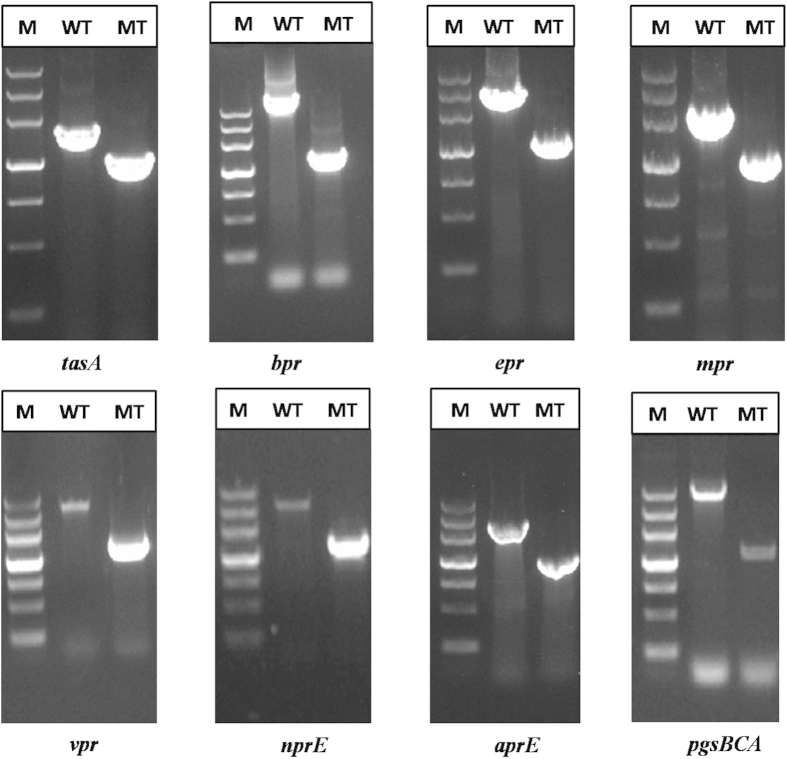
Confirmation of gene deletions via PCR. Chromosomal DNA served as the template for amplification. Lane M: DNA marker III; Lane WT: strains amplified with relevant detection primers using *B. amyloliquefaciens* NK-1 chromosomal DNA as the template; Lane MT: strains amplified with the relevant detection primers using chromosomal DNA from the gene deletion strains’ as the template.

We constructed these gene deletion mutants via a marker-less knockout method. This method is based on using the *upp* gene, which encodes uracil-phosphoribosyl-transferase, as the counter-selectable marker. The *upp* cassette and 5-fluorouracil (5-FU) selection were used to identify marker-less gene deletions^29^. According to the PCR results shown in Fig. 2 and the DNA sequencing results, we confirmed that the gene mutant strains had been successfully constructed.

### The effects of *tasA* gene deletion on biofilm formation

**Figure 3:**
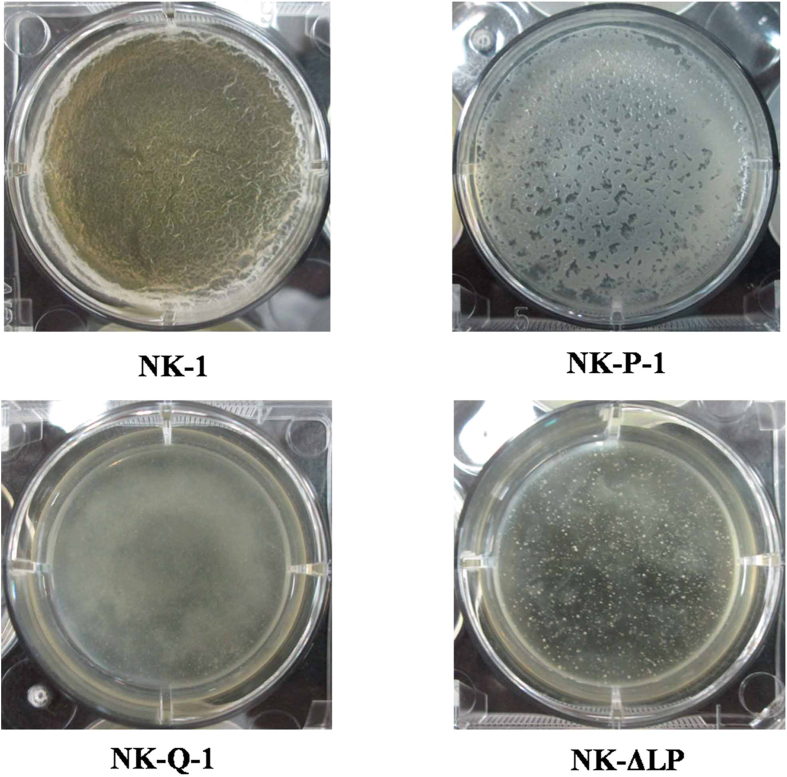
Cell pellicle formation in the *B. amyloliquefaciens* NK-1, *B. amyloliquefaciens* NK-P-1, *B. amyloliquefaciens* NK-Q-1 and *B. amyloliquefaciens* NK-ΔLP strains. Cells were cultured at 30 °C for 72 h in MSgg broth in a six-well microtiter dish.

Our previous work showed that *B. amyloliquefaciens* NK-1 could form structurally complex biofilms^30^. Genetic, biochemical and cytological evidences suggested that this complex extracellular matrix is mainly composed of TasA and Eps and the absence of TasA or Eps results in a residual matrix^17^,^31^. Levan and γ-PGA also contribute to bacterial biofilm formation, cross-linking with other components to make the biofilm complete^32^,^33^. Biofilm formation by the NK-1, NK-P-1, NK-Q-1 and NK-ΔLP strains was observed and the results were shown in Fig. 3. As expected, the NK-1 strain was able to form a complete pellicle, whereas the *tasA* gene deletion NK-P-1 strain could only form a deficient, incomplete pellicle. Unexpectedly, neither of the *pgsBCA* genes deletion strains (NK-Q-1 and NK-ΔLP) could form a pellicle. This result indicates that γ-PGA is the main component of the biofilm formed by the γ-PGA-producing strain and that the strain with deficient γ-PGA production could not form a biofilm.

### Comparison of γ-PGA production beween B. amyloliquefaciens NK-1 and the mutant NK-P-X strains in flask culture

**Figure 4:**
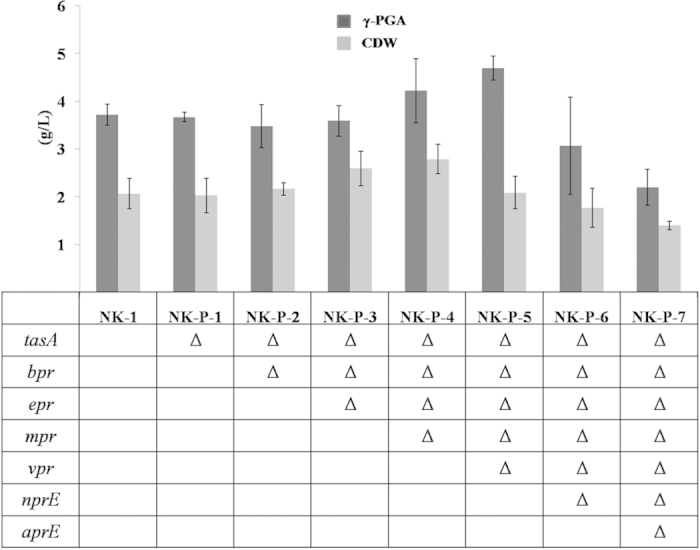
γ-PGA fermentation results in the *B. amyloliquefaciens* NK-1 and mutant NK-P-X strains after 48 h of cultivation. Values represent the means ± SD. Asterisks indicate significant difference from the NK-1 strain (P < 0.05). All cultures were repeated at least five times.

The effects of the deletion of *tasA* and the six-extracellular protease genes on γ-PGA production were characterized in this work. The γ-PGA fermentation results obtained from *B. amyloliquefaciens* NK-1 and the gene deletion mutant strains are shown in Fig. 4. γ-PGA production remained unchanged after deleting the *tasA, bpr, epr* and *mpr* genes. However, the NK-P-5 strain, harboring further deletion of the *vpr* gene, showed increased γ-PGA production. The NK-P-5 strain exhibited the highest γ-PGA yield, which was a 24.2% increase compared with the NK-1 strain, leading to a titer of 4.62 g/L, compared with 3.72 g/L for the control. γ-PGA production was lower in the strains in which the *nprE* and *aprE* genes were deleted. γ-PGA production from the NK-P-7 strain was 2.19 g/L, which was 47.4% lower than in the NK-P-5 strain. Moreover, the dry cell weight (DCW) of the NK-P-7 strain was lower than other strains, and this strain was observed to enter the cell decline phase earlier.

### Comparison of levan production between the B. amyloliquefaciens NK-ΔLP and mutant NK-Q-X strains in flask culture

**Figure 5:**
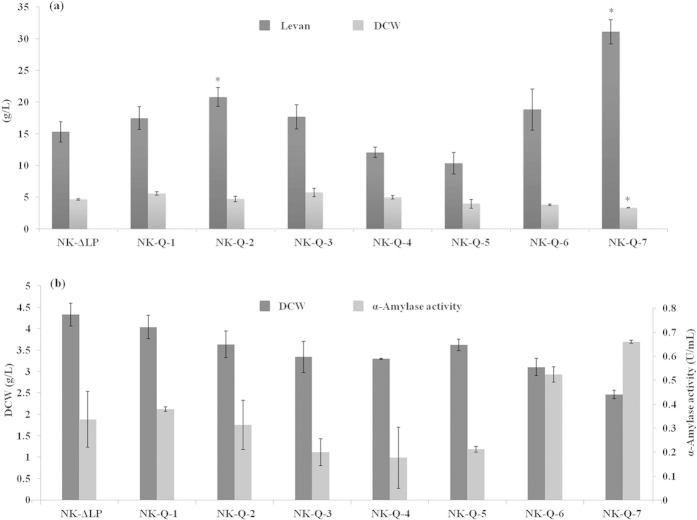
Levan and α-amylase fermentation results in the *B. amyloliquefaciens* NK-ΔLP and mutant NK-Q-X strains. (**a**) Comparison of levan production and dry cell weights between NK-ΔLP and the mutant strains after 48 h of cultivation. (**b**) Comparison of α-amylase production and dry cell weights between NK-ΔLP and the mutant strains after 48 h of cultivation. Values represent the means ± SD. Asterisks indicate significant difference from the NK-ΔLP strain (P < 0.05). All cultures were repeated at least five times.

To evaluate the effect of the accumulation of gene-targeted *B. amyloliquefaciens* mutants on levan production, flask culture of *B. amyloliquefaciens* NK-ΔLP and the mutant strains was undertaken under identical conditions. We sought to delete the *tasA* gene for two reasons: (1) disruption of *tasA* can conserve energy and favor the production of levan via the metabolic flux; (2) Cells in biofilms are embedded in the extracellular matrix, which contains TasA, the major matrix protein. We speculated that by removing TasA, which is known to be bound to cells^17^, we may influence the export of extracellular levansucrase. Therefore it might have been possible that lack of TasA increases the production of levan. The fermentation results shown in Fig. 5a indicated that the deletion of *tasA* gene did affect levan production. The NK-Q-1 strain showed a slight 14.4% increase in levan production (17.5 g/L) compared with the NK-ΔLP strain (15.3 g/L).

Levan is synthesized in the medium by secreted levansucrase. We reasoned that the lack of extracellular proteases might make the extracellular levansucrase more stable, thereafter improving levan production in the medium. As shown in Fig. 5a, the extracellular protease gene deletion strains NK-Q-2 and NK-Q-7 exhibited levan production increase. Among these strains, NK-Q-7 displayed the highest increase in productivity (31.1 g/L), resulting in 103% improvement of levan production compared with the NK-ΔLP control. The molecular weight of levan obtained from NK-Q-7 strain was 4, 600 Da, and its purity reached 94.1 ± 1.3%. However, not all of the mutant strains showed improved levan production; the NK-Q-4 and NK-Q-5 strains displayed decreases of 21.3% and 32.4% in levan production, respectively.

### Comparison of α-amylase production between B. amyloliquefaciens NK-ΔLP and the mutant NK-Q-X strains in flask culture

To evaluate the effect of the gene deletions on extracellular protein production, we also determined the α-amylase production. The results regarding α-amylase production are shown in Fig. 5b. Consistent with the levan fermentation results (Fig. 5a), the NK-Q-7 strains showed significantly increased α-amylase production to approximately 0.66 U/mL of amylase activity, which was 94.1% higher compared with the NK-ΔLP strain.

### Determination of extracellular protease activities in the *B. amyloliquefaciens* NK-Q-X strains

We further analyzed extracellular protease activities in the NK-Q-X strains at the end of stationary phase during the levan fermentation process, and the results are presented in Table 1. The protease activities of NK-ΔLP and NK-Q-1 were comparable, indicating that the deletion of *tasA* had no effect on cell protease activity. The *bpr* gene deletion strain NK-Q-2 showed a 38% decrease compared with the NK-ΔLP strain, whereas the NK-Q-3 strain, in which both *bpr* and *epr* were deleted, exhibited an approximately a 50% decrease in protease activities. Surprisingly, and in contrast to previous studies, the detected protease activities was unchanged after deletion the *mpr* and *vpr* genes. However, the strain without *nprE* and *aprE* gene showed further decreases in protease activity; protease activity was decreased by 56% in NK-Q-6 and by a notable 86% in NK-Q-7 compared with the NK-ΔLP strain.

## Discussion

**Figure 6:**
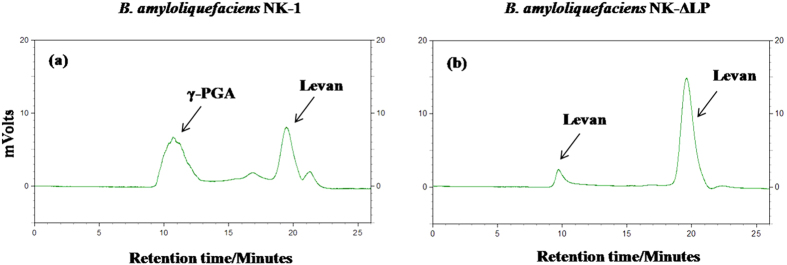
GPC results for γ-PGA and levan products. (**a**) GPC spectrum of γ-PGA obtained from *B. amyloliquefaciens* NK-1 and (**b**) GPC spectrum of levan obtained from *B. amyloliquefaciens* NK-ΔLP.

*B. amyloliquefaciens* NK-1 is derived from the LL3 strain, which was isolated from fermented food (Korea bibimbap paste)^9^, thus it is safe for human beings. In our previous work, we found that the NK-1 strain could co-produce γ-PGA and levan simultaneously (Fig. 6a). After deleting the *pgsBCA* cluster, the obtained NK-ΔLP strain could not produce γ-PGA, and the remaining product mainly consisted of levan, at a purity reaching 92.7% (Fig. 6b) ^7^. From our previously work, we determined that the bacteria could produce levan products of two different molecular weights. Higher-molecular-weight levan is produced at early timepoints and is then hydrolyzed to lower-molecular-weight levan by levanase, and the low-molecular-weight levan is dominant after 48 h of fermentation^7^. The levan molecular weight obtained from the NK-ΔLP strain was mostly around 5 kDa^7^, which is significantly lower than other reported levan products^8^. However, levan production needs to be improved to meet the requirements for industrial application^34^.

We deleted the *tasA* gene from the NK-1 strain, and the resulting NK-P-1 strain could form incomplete biofilms, whereas the *tasA* and *pgsBCA* double-deletion NK-Q-1 strain could not form a biofilm (Fig. 3). We further studied the effects of these deletions on levan synthesis. The NK-Q-1 strain showed a 14.4% increase in levan production compared with the NK-ΔLP strain (Fig. 5a). Unlike our expected, the deletion of *tasA* gene had little effect on levan production.

*Bacillus* species can produce high levels of extracellular proteases to degrade secreted heterologous proteins^19^. Many protease-deficient strains have been constructed and display favorable features for the improvement of heterologous protein production. *B. subtilis* WB600 is a strain that is deficient in six-extracellular-protease (Δ*nprE*, Δ*nprB*, Δ*aprE*, Δ*epr*, Δ*mpr*, Δ*bpr*) and showes improved production of heterologously expressed β-lactamase, streptokinase and the antidigoxin single-chain antibody fragment over the wild-type strain^35^,^36^,^37^. Although many works have focused on the effect of extracellular-protease-deficiency on heterologous proteins, few efforts have been made to study its effect on endogenous protein production. We hypothesized that the extracellular proteases degrade not only misfolded proteins but also degraded proteins (which still exhibit low catalytic activity) or even fully-functional proteins in the end stage of fermentation when nitrogen is limited. Based on this hypothesis, we aimed to improve levan production by knocking out the microbe’s extracellular protease genes. Levan is produced by the secreted levansucrase SacB, and extracellular proteases may affect the amount and activity of SacB to some degree, decreasing levan production. And the extracellular-proteases-deficient strains probably exhibit increased production of extracellular protein including SacB, thereafter increase the production of levan. The NK-1 strain exhibits seven extracellular proteases: Bpr, Epr, Mpr, Vpr, NprE, AprE and WprA^38^, and we aimed to delete all of these associated genes. However, we failed to delete the *wprA* gene for unknown reasons. The other six genes were successfully deleted.

The six-gene-deletion NK-Q-7 strain showed the highest levan production, presenting increases of approximately about 102% and 78% compared with the NK-ΔLP and NK-Q-1 strains, respectively. We speculated that the increase of levan production was related to the increase of SacB production, and the deletion of extracellular protease genes improved the production of extracellular protein-SacB. To evaluate our speculation, we further determined the effect of gene deletions on another endogenous product α-amylase (a secreted *Bacillus* protein). The NK-Q-7 strain showed the highest yield of α-amylase as well, with production being increased by 96% and 74%, compared with the NK-ΔLP and NK-Q-1 strains, respectively. These results demonstrate that the deficiency of extracellular protease genes indeed increase the cells’ production of their own secreted proteins. Despite exhibiting highest production, the DCW of NK-Q-7 was the lowest among these strains. Additionally, more frequent cell lysis was detected in the end stage of fermentation and the number of living cells was 28 ± 9% less than that of the control NK-ΔLP strain. Another interesting phenomenon observed in this strain was the shorter time (24 h) required for the organism to adapt to the high carbon source conditions (in the levan seed culture) compared with other six strains (30–36 h) when transferred from incubation in LB medium. This particular feature might offset the early cell lysis in the end stage of fermentation.

We also characterized the extracellular protease activity of the NK-Q-X strains in levan fermentation medium. Consistent with our hypothesis, the extracellular protease activity of the NK-Q-7 strain showed a dramatic decrease to approximately 14.3% of that in the NK-1 strain (Table 1). However, this extracellular protease activity was still higher than in other previously reported strains, such as *B. subtilis* GB2054 (with inactivation of two extracellular proteases, NprE and Apr, and no detectable extracellular protease activity)^39^ and *B. subtilis* WB700 (with inactivation of seven extracellular proteases, NprE, NprB, AprE, Epr, Mpr, Bpr and Vpr, and decreased extracellular protease activity to approximately 0.14% of that in the wild-type strain)^40^. This difference may be attributable to the applied cell culture conditions. However, not all of the mutant strains showed increased levan production. For example, the NK-Q-4 and NK-Q-5 strains were deficient in extracellular protease activity, yet showed reduced levan production. The *mpr* and *vpr* gene deletions might be harmful to levan production.

As the production of TasA and extracellular proteases consumes a great deal of energy, we also studied the effect of these gene deletions on γ-PGA production. However, their deletions had little effect on γ-PGA production, and only the NK-P-5 strain exhibited a slight increase in γ-PGA production (Fig. 4).

In summary, we proposed a new strategy for improving levan production in this work. We deleted the *tasA* gene, six extracellular protease genes and the *pgsBCA* cluster. The resulting NK-Q-7 strain produced 31.1 g/L levan, which was 103% higher than the production in the NK-ΔLP control strain. The NK-Q-7 also yielded 96% more α-amylase. These results indicate that NK-Q-7 strain could be utilized as a candidate cell factory for secreted protein production or secreted protein related product synthesis.

## Methods

### Strains, plasmids and growth conditions

All of the strains and plasmids used in this work are listed in Table 2. The *B. amyloliquefaciens* NK-1 strain was a derivative of the LL3 strain with the endogenous plasmid pMC1 and the *upp* gene deleted^41^,^42^. The *B. amyloliquefaciens* NK-ΔLP strain was a derivative of the NK-1 strain with the *pgsBCA* cluster deleted. *Escherichia coli* DH5α was used for plasmid propagation and transformation. The *dam-*and *dcm-* deficient *E. coli* strain GM2163 was used for plasmid demethylation.

For routine strain construction and maintenance, the *B. amyloliquefaciens* and *E. coli* strains were grown at 37 °C in Luria-Bertani (LB) medium. To produce γ-PGA, the *B. amyloliquefaciens* strains were cultured at 37 °C and 180 rpm, for 48 h in γ-PGA fermentation medium^43^. To produce levan, *B. amyloliquefaciens* strains were cultured at 37 °C and 180 rpm, for 48 h in levan fermentation medium (pH 6.0), which containing 250.9 g/L sucrose, 2.6 g/L urea, 0.62 g/L MgSO_4_, 8.16 g/L KH_2_PO_4_, 18.24 g/L K_2_HPO_4_·3H_2_O, 1 mM FeSO_4_, 1 mM CaCl_2_, 1 mM MnSO_4_ and 1 mM ZnCl_2_. To produce α-amylase, the *B. amyloliquefaciens* strains were cultured at 37 °C and 180 rpm, for 48 h in α-amylase fermentation medium (pH 7.0), containing 10 g/L soluble starch, 2 g/L tryptone, 1 g/L KH_2_PO_4_, 2.5 g/L Na_2_HPO_4_, 2 g/L (NH_4_)_2_SO_4_, 1 g/L NaCl, 0.05 g/L MgSO_4_·7H_2_O and 0.05 g/L CaCl_2_^44^.

The concentrations of the antibiotics used in this work were as follow: 100 μg/mL ampicillin, 5 μg/mL chloramphenicol. The final concentration of 5-FU was 100 μg/mL.

### DNA manipulation and plasmid construction

To construct the gene deletion vectors, the temperature-sensitive pKSU plasmid was used, which was derived from the pKSV7 plasmid^45^,^46^. The *upp* expression cassette was ligated to the pKSV7 plasmid as a reverse selection marker. All of the oligonucleotide primers used in this work are listed in Table 3. We decided to delete eight genes: *tasA*, *aprE*, *bpr*, *epr*, *mpr*, *nprE*, *vpr* and *pgsBCA*. The upstream and downstream fragments of the genes targeted for deletion were amplified with PrimeSTAR HS DNA polymerase (Takara Bio, Japan) using the primers N-SF/N-SR and N-XF/N-XR, respectively (N represents the relevant gene name). The obtained upstream and downstream DNA fragments were joined via overlap-PCR. The combined fragments were first ligated to the pMD19-T simple vector (Takara Bio, Japan). All of the constructed plasmids were verified through DNA sequencing (BGI, China). They were then restricted by endonucleases *Sal* I and *Bam*H I and ligated into the pKSU vector digested with the same enzymes, respectively. Finally the generated gene deletion plasmids were designated pKSV7-ΔN.

### Construction of gene knockout mutant strains

To carry out multiple gene deletions in a single strain, a modified marker-less gene deletion method was used to construct the gene knockout mutant strains^29^,^46^. The primers N-SS and N-XX (N represents the relevant gene name) were used to determine the gene deletion mutants via PCR. We deleted the *tasA*, *bpr*, *epr*, *mpr*, *vpr*, *nprE* and *aprE* genes in turn from the NK-1 strain. The gene disruption mutants were designated *B. amyloliquefaciens* NK-P-1, NK-P-2, NK-P-3, NK-P-4, NK-P-5, NK-P-6 and NK-P-7, respectively. We further deleted the *pgsBCA* cluster (for γ-PGA synthesize) from the NK-P-X strains (X represents the numbers 1–7). The resulting seven γ-PGA-deficient strains were designated NK-Q-X.

### Production of γ-PGA, levan and α-amylase in flask culture

For γ-PGA production, single colonies of the *B. amyloliquefaciens* NK-P-X strains were transferred to 50 mL of γ-PGA fermentation medium. After 18 h of incubation at 37 °C and 180 rpm, l mL of the cultures was transferred to 100 mL of fermentation medium in shaking flasks and then fermented for 48 h.

The *B. amyloliquefaciens* NK-Q-X strains were used for levan and α-amylase production. As the cell growth rates in the levan fermentation medium and the α-amylase fermentation medium were lower than in the γ-PGA fermentation medium, the seed culture times were extended to 24 h–40 h. All cultures were repeated at least five times.

### Enzyme assays

α-amylase activity was measured via a modified dinitrosalicylic acid (DNS) method, which was based on the amount of reducing sugars released from soluble starch^47^. A mixture (pH 5.9) containing 1% soluble starch and 0.05 M sodium citrate buffer was heated at 40 °C for 10 min in a water bath. Then, 0.1 mL of culture extract was added to the substrate and incubation was continued at 40 °C for 30 min with gentle shaking. The reaction was subsequently stopped by adding 2 mL of the DNS reagent, after which the reaction was heated at 100 °C for 10 min, and its absorbance was measured at 540 nm. A glucose standard curve was used to determine α-amylase activity. One unit (U) of α-amylase activity was defined as the amount of enzyme that liberated one micromole of reducing sugar in one minute under the assay conditions.

Protease activity in the supernatant of the levan fermentation medium at the end of the stationary phase was measured via a modified casein digestion method^48^. One milliliter of 1% casein solution in 0.2 M Tris buffer (pH 8.5) was incubated with 1 mL of test culture for 30 min at 37 °C. The reaction was stopped by the addition of 2 mL of 10% trichloroacetic acid (TCA) solution. After centrifugation at 1000 rpm for 5 min, 1 mL of the supernatant was reacted with the Folin-reagent (Dingguo, China). The protease activity was determined from the absorbance of the reaction at 680 nm. The protease activity determined for NK-ΔLP in levan fermentation medium was defined as 100%.

### Biofilm formation

For the analysis of pellicle formation, the NK-1, NK-P-1, NK-Q-1 and NK-ΔLP strains were cultured on LB agar for 18 h and subsequently incubated in LB broth to an OD_600_ of 1.0. Next, 10 μL aliquots of the cultures were added to 10 mL of MSgg broth^17^ in a six-well microtitre dish. The dishes were incubated at 30 °C for 72 h without agitation, and the pellicles that formed were photographed by a digital camera equipped with a close-up lens (Canon, Tokyo, Japan).

### Analytical procedures

The optical density (OD) of the cultures was measured with a SHIMADZU UV-1800 spectrophotometer (Kyoto, Japan). The dry cell weight was determined from 100 mL of broth, and the cells were harvested via centrifugation and dried at 50 °C for 24 h to a constant weight after washed with distilled water. γ-PGA was purified by a previously described method^9^,^49^. The procedure for the purification of levan was similar to that for γ-PGA, with the exception of the use a 3500 MW dialysis bag following the precipitated of levan with four-fold volumes the cold ethanol. The molecular weight of the levan was determined by a gel permeation chromatography (GPC) system^42^. An Alltech system controller (Alltech Associates Inc., US) with a Shodex KW804 column (Showa Denko KK, Japan) and a refractive index (RI) detector (Schambeck SFD GmbH, Germany) were used. 0.25 mol/L NaNO_3_ was used as the mobile phase with a flow rate of 0.6 mL/min. Shodex Pullulan-82 standards were used to construct the calibration curve. To measure the levan product purity, 50 mg levan product was hydrolyzed by 6 mol/L HCl at 100 °C for 15 min. The optical density at 291 nm was measured by a SHIMADZU UV-1800 spectrophotometer (Kyoto, Japan) to determine the concentration of fructose in the hydrolyzate^50^. The fructose standards were used to construct the calibration curve. The number of the monosaccharide (N) in levan product was defined as: 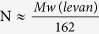 The purity of levan (%) = fructose concentration 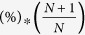. The living cells were detected by the optical microscope (XSP-8CA, Shanghai, China) and the methylene blue dye after 48 h of cultivation.

## Additional Information

**How to cite this article**: Feng, J. *et al.* Recruiting a new strategy to improve levan production in *Bacillus amyloliquefaciens*. *Sci. Rep.* **5**, 13814; doi: 10.1038/srep13814 (2015).

## Figures and Tables

**Figure 1:**
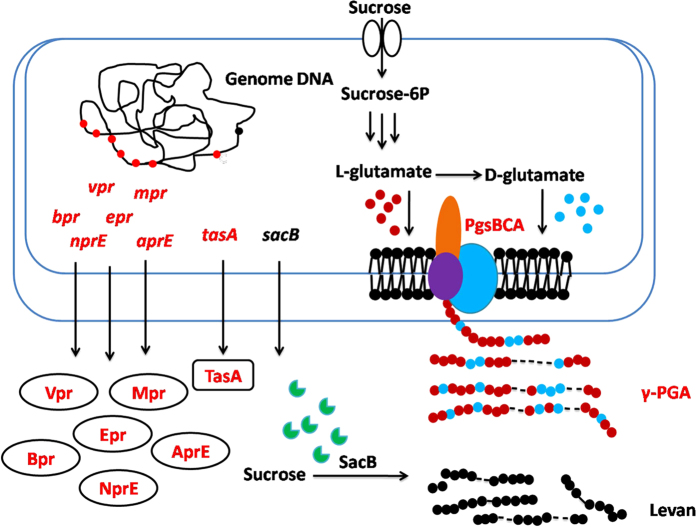
Metabolic pathways associated with levan biosynthesis in *Bacillus amyloliquefaciens* and engineering strategies for levan production. The red font indicates the genes deleted in this study and the corresponding deficient products. Metabolite symbols: Sucrose-6P, sucrose-6-phosphate; Bpr, bacillopeptidase F; Epr, extracellular serine protease; Mpr, extracellular metalloprotease; Vpr, extracellular serine protease; NprE, extracellular neutral metalloprotease; AprE, extracellular alkaline serine protease; TasA, major biofilm matrix protein; *pgsBCA*, poly-γ-glutamate synthase.

**Table 1:** Comparison of extracellular protease activity between the *Bacillus amyloliquefaciens* NK-ΔLP and *Bacillus amyloliquefaciens* NK-Q-X strains in levan fermentation medium.

Strains	Extracellular protease activity (%)
NK-ΔLP	100 ± 12.3
NK-Q-1	104 ± 1.7
NK-Q-2	62.1 ± 3.3
NK-Q-3	50.5 ± 1.4
NK-Q-4	55.6 ± 17.4
NK-Q-5	54.6 ± 2.0
NK-Q-6	44.2 ± 0.7
NK-Q-7	14.3 ± 1.2*

All cultures were repeated at least five times.

Asterisks indicate significant difference from the NK-ΔLP strain (P < 0.05).

**Table 2:** Strains and plasmids used in this study.

Strains and plasmids	Relevant genotype and characteristics	Source
**Strains**		
* B. amyloliquefaciens* LL3	wild type	(9)
* B. amyloliquefaciens* NK-ΔLP	NK-1 derivative, Δ*pgsBCA*	This lab
* B. amyloliquefaciens* NK-1	LL3 derivative, ΔpMC1, Δ*upp*	(42)
* B. amyloliquefaciens* NK-P-1	NK-1 derivative, Δ*tasA*	This work
* B. amyloliquefaciens* NK-P-2	NK-1 derivative, Δ*tasA*, Δ*bpr*	This work
* B. amyloliquefaciens* NK-P-3	NK-1 derivative, Δ*tasA*, Δ*bpr*, Δ*epr*	This work
* B. amyloliquefaciens* NK-P-4	NK-1 derivative, Δ*tasA*, Δ*bpr*, Δ*epr*, Δ*mpr*	This work
* B. amyloliquefaciens* NK-P-5	NK-1 derivative, Δ*tasA*, Δ*bpr*, Δ*epr*, Δ*mpr*, Δ*vpr*	This work
* B. amyloliquefaciens* NK-P-6	NK-1 derivative, Δ*tasA*, Δ*bpr*, Δ*epr*, Δ*mpr*, Δ*vpr*, Δ*nprE*	This work
* B. amyloliquefaciens* NK-P-7	NK-1 derivative, Δ*tasA*, Δ*bpr*, Δ*epr*, Δ*mpr*, Δ*vpr*, Δ*nprE*, Δ*aprE*	This work
* B. amyloliquefaciens* NK-Q-1	NK-P-1 derivative, Δ*pgsBCA*	This work
* B. amyloliquefaciens* NK-Q-2	NK-P-2 derivative, Δ*pgsBCA*	This work
* B. amyloliquefaciens* NK-Q-3	NK-P-3 derivative, Δ*pgsBCA*	This work
* B. amyloliquefaciens* NK-Q-4	NK-P-4 derivative, Δ*pgsBCA*	This work
* B. amyloliquefaciens* NK-Q-5	NK-P-5 derivative, Δ*pgsBCA*	This work
* B. amyloliquefaciens* NK-Q-6	NK-P-6 derivative, Δ*pgsBCA*	This work
* B. amyloliquefaciens* NK-Q-7	NK-P-7 derivative, Δ*pgsBCA*	This work
* E. coli* DH5α	F^−^, φ80d*lac*ZΔM1, Δ(*lacZYA-argF*)U169, *deoR*, *recA*1, *endA*1, *hsdR*17(r_k_^−^, m_k_^+^), *phoA*, *supE*44, λ^−^ *thi*-1, *gyrA*96, *relA*1	This lab
* E. coli* GM2163	F^−^, *ara-14 leuB6 thi-1 fhuA31 lacY1 tsx-78 galK2 galT22 supE44 hisG4 rpsL 136 (Str*^*r*^*) xyl-5 mtl-1 dam13::*Tn9 (Cam^r^) *dcm-6 mcrB1 hsdR2 mcrA*	This lab
**Plasmids**		
* *pKSV7	Shuttle vector, temperature-sensitive (ts) replication origin, Amp^r^ (gram-negative) and Cm^r^ (gram-positive)	(45)
* *p-KSU	pKSV7-derivation with *upp* gene	(46)
* *pKSV7-△LP	p-KSU-derivation with deletion fragment of *pgs operon*	This lab
* *pKSV7-△tasA	p-KSU-derivation with deletion fragment of *tasA*	This work
* *pKSV7-△bpr	p-KSU-derivation with deletion fragment of *bpr*	This work
* *pKSV7-△epr	p-KSU-derivation with deletion fragment of *epr*	This work
* *pKSV7-△mpr	p-KSU-derivation with deletion fragment of *mpr*	This work
* *pKSV7-△vpr	p-KSU-derivation with deletion fragment of *vpr*	This work
* *pKSV7-△nprE	p-KSU-derivation with deletion fragment of *nprE*	This work
* *pKSV7-△aprE	p-KSU-derivation with deletion fragment of *aprE*	This work

**Table 3:** Primers used in this study.

Primers	Sequence (5′–3′)
tasA-SF	CCC*GGATCC*ACTCTCAAAATACATCAGACAAATAG
tasA-SR	CGTTCAGGAACGTTCTTGCTTTTTTGCTGTCTAATGTTTC
tasA-XF	ACAGCAAAAAAGCAAGAACGTTCCTGAACGATAATACATC
tasA-XR	GGG*GTCGAC*GAATTTTTTCGCATGTTCAAACATT
tasA-SS	GACTGACGTCATGAGCTGCTGGGTTTTT
tasA-XX	CCAAGTTCTTTTTCACCGGGAACGCC
bpr-SF	CCCC*GGATCC*TAACGCCCTTAAAACGAAATCT
bpr-SR	TTATTTTTCACATTTCTTTTTCTTTTTCATAGTCTGCCTC
bpr-XF	ATGAAAAAGAAAAAGAAATGTGAAAAATAACAAGAC
bpr-XR	CCCC*GTCGAC*TTACTGAACGTCACTCATATC
bpr-SS	TAGACACGTATTTTCAGCGTGATCC
bpr-XX	GCTCGGAGGCTATTCAGTTGCGTAT
epr-SF	CGC*GGATCC*CCAGGGATGGACAAGAAC
epr-SR	TAAGCGCTCGTATTCGTTCTCGTTACTGCAGG
epr-XF	CAGTAACGAGAACGAATACGAGCGCTTATTGG
epr-XR	AGGC*GTCGAC*AAAGCGGAGGAGAAATACAG
epr-SS	GCGGGTTTATCCTGTTCTTAATCGG
epr-XX	GGCACCGTTATTTTCTACAGCCTGG
mpr-SF	CCC*GGATCC*ACTCTCAAAATACATCAGACAAATAG
mpr-SR	CGTTCAGGAACGTTCTTGCTTTTTTGCTGTCTAATGTTTC
mpr-XF	ACAGCAAAAAAGCAAGAACGTTCCTGAACGATAATACATC
mpr-XR	GGG*GTCGAC*GAATTTTTTCGCATGTTCAAACATT
mpr-SS	GACTGACGTCATGAGCTGCTGGGTTTTT
mpr-XX	CCAAGTTCTTTTTCACCGGGAACGCC
vpr-SF	CCCC*GGATCC*TAACGCCCTTAAAACGAAATCT
vpr-SR	TTATTTTTCACATTTCTTTTTCTTTTTCATAGTCTGCCTC
vpr-XF	ATGAAAAAGAAAAAGAAATGTGAAAAATAACAAGAC
vpr-XR	CCCC*GTCGAC*TTACTGAACGTCACTCATATC
vpr-SS	TAGACACGTATTTTCAGCGTGATCC
vpr-XX	GCTCGGAGGCTATTCAGTTGCGTAT
nprE-SF	CCCC*GGATCC*TAACGCCCTTAAAACGAAATCT
nprE-SR	TTATTTTTCACATTTCTTTTTCTTTTTCATAGTCTGCCTC
nprE-XF	ATGAAAAAGAAAAAGAAATGTGAAAAATAACAAGAC
nprE-XR	CCCC*GTCGAC*TTACTGAACGTCACTCATATC
nprE-SS	TAGACACGTATTTTCAGCGTGATCC
nprE-XX	GCTCGGAGGCTATTCAGTTGCGTAT
aprE-SF	CCCC*GGATCC*TAACGCCCTTAAAACGAAATCT
aprE-SR	TTATTTTTCACATTTCTTTTTCTTTTTCATAGTCTGCCTC
aprE-XF	ATGAAAAAGAAAAAGAAATGTGAAAAATAACAAGAC
aprE-XR	CCCC*GTCGAC*TTACTGAACGTCACTCATATC
aprE-SS	TAGACACGTATTTTCAGCGTGATCC
aprE-XX	GCTCGGAGGCTATTCAGTTGCGTAT

The restriction enzyme cleavage sites are underlined.
